# Outcomes of Reduction Mammaplasty in Adolescents vs Average-Age Patients: A 3-Year Single-Center Retrospective Analysis

**DOI:** 10.31486/toj.23.0079

**Published:** 2024

**Authors:** H. Harvak Hajebian, Salomon Puyana, Christopher R. Babycos, Michael T. Friel

**Affiliations:** ^1^ Department of Plastic and Reconstructive Surgery, Ochsner Clinic Foundation, New Orleans, LA; ^2^ Division of Plastic Surgery, Department of Surgery, Tulane University School of Medicine, New Orleans, LA; ^3^ The University of Queensland Medical School, Ochsner Clinical School, New Orleans, LA

**Keywords:** *Adolescent*, *macromastia*, *mammaplasty*

## Abstract

**Background:** The onset of macromastia symptomatology occurs most often at puberty, yet most females undergo breast reduction surgery during the fifth decade of life. Adolescent patients with macromastia may benefit from reduction mammaplasty, yet outcome data are limited to a small number of institutions.

**Methods:** We conducted a retrospective medical records review of all patients who underwent reduction mammaplasty at our institution during the years 2016 to 2019. Patients were divided into 2 cohorts based on age: adolescent (10 to 24 years) and average age (≥44 years). Demographics and outcome measures were collected from follow-up evaluations at 1-week, 1-month, 3-month, 6-month, and 12-month intervals postoperatively.

**Results:** A total of 141 patients met the inclusion criteria for the study. Mean age at surgery was 19 ± 3.2 years in the adolescent group and 53 ± 7.4 years in the average-age group. No significant differences in complications related to wound healing (42.9% vs 50.0%, *P*=0.418) or total postoperative complications (18.4% vs 19.6%, *P*=0.863) were found between adolescent and average-age patients, respectively.

**Conclusion:** Complications related to wound healing are common in reduction mammaplasty, although rates of life-threatening complications are rare. In this 3-year review comparing the outcomes of adolescent vs average-age patients who underwent reduction mammaplasty at the same institution, no significant differences in postoperative complication rates were found. Our data suggest that adolescent patients with macromastia should not defer reduction mammaplasty out of concern for higher complication rates because of age alone.

## INTRODUCTION

Reduction mammaplasty has been shown to improve dermatologic, musculoskeletal, and psychosocial symptoms of macromastia and may lead to an overall increase in quality of life.^1,2^ Although the precise etiology of macromastia is not entirely understood, most females report the onset of symptoms during initial breast development at puberty.^3,4^ In efforts to reduce the burden of excessive breast tissue, patients frequently attempt provider-recommended physical therapy and scheduled exercise, yet the outcomes data on nonoperative management of macromastia have been disappointing.^5,6^ While the reasoning is unclear, the majority of females do not undergo reduction mammaplasty until the fifth decade of life.^7^

Reduction mammaplasty is not considered a high-risk procedure, but complication rates have been reported as high as 50% to 53.9% in adult patients.^8,9^ Although classification criteria have yet to be established, the most common complications following reduction mammaplasty are minor and related to wound healing, such as wound dehiscence, scarring, altered sensation of the breast/nipple-areolar complex, hematoma/seroma, and infection.^5,7-9^ However, for adolescent patients, defined in 2018 as between 10 years and 24 years of age,^10^ data on complications are lacking, with data predominantly limited to works from one institution.^2,11,12^ Furthermore, studies with robust sample sizes comparing complication rates between adolescents and average-age patients (those ≥44 years) within the same institution are deficient in the literature. As such, the recommendation for early breast reduction in young women and girls remains controversial.

In this study, we compared the outcomes between adolescent patients and average-age patients who underwent reduction mammaplasty at a single institution.

## METHODS

**Figure 1.**
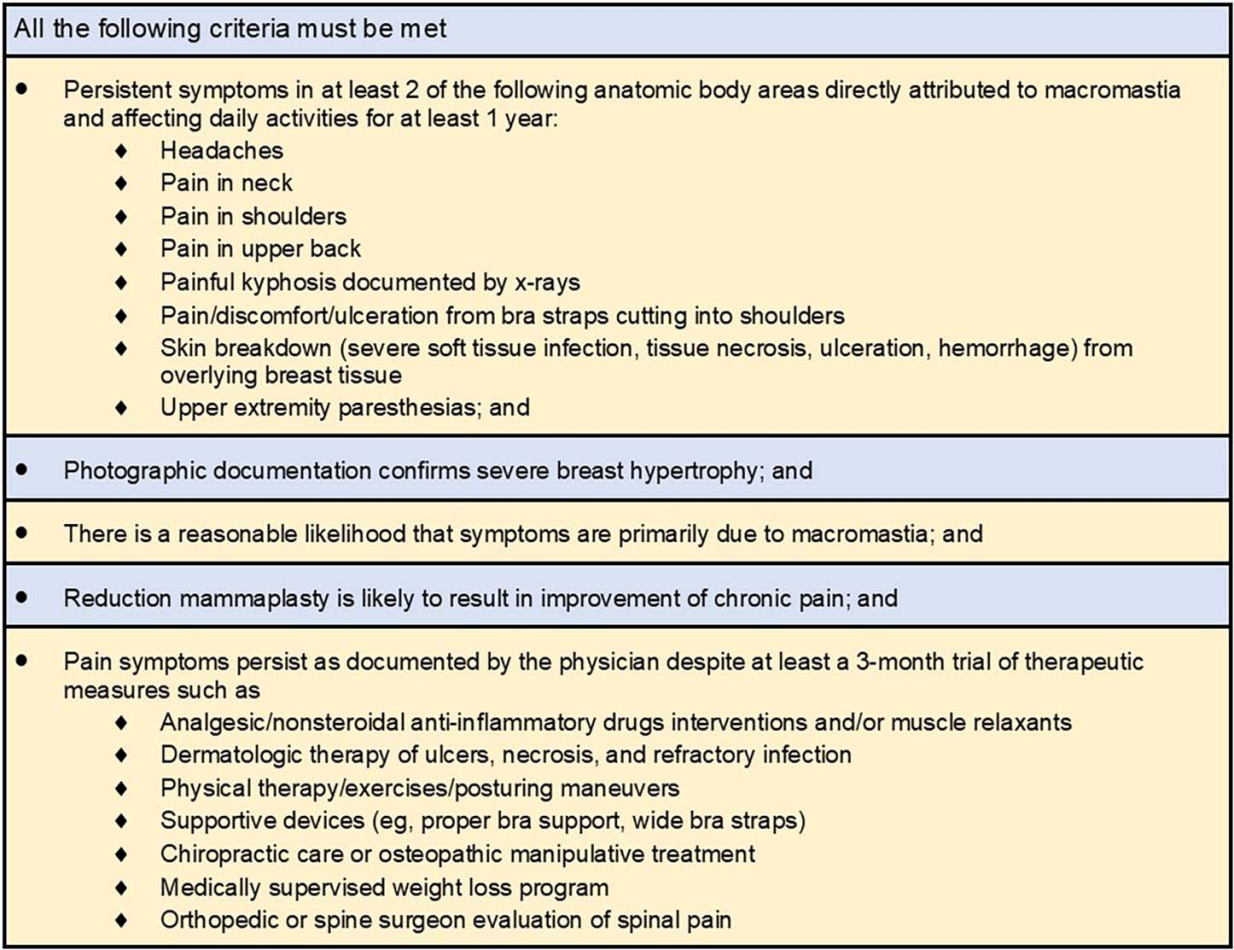
American Society of Plastic Surgeons criteria for surgically necessary treatment of macromastia.^13^

**Figure 2.**
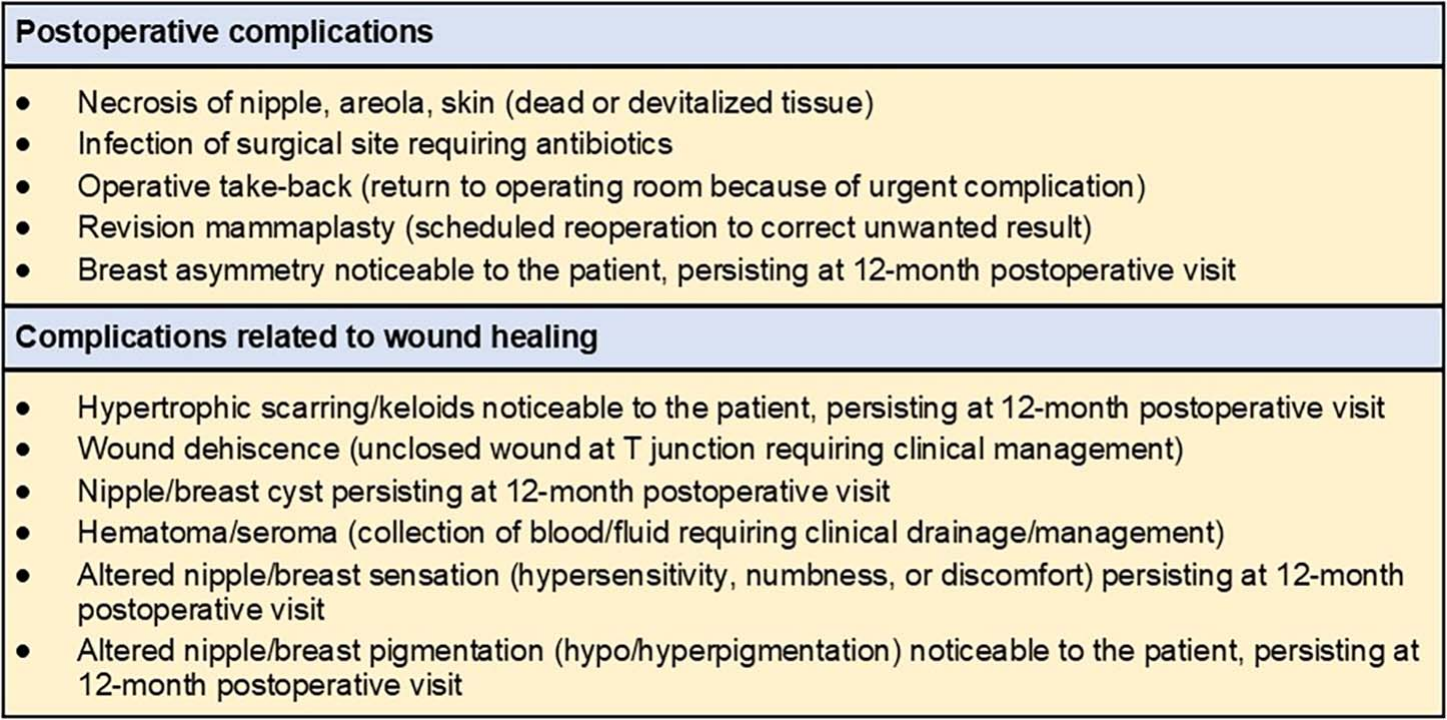
American Society of Plastic Surgeons list and definitions of postoperative complications.^14^

After obtaining institutional review board approval (2021.128), we retrospectively reviewed our institution's database of female patients aged 10 to 24 years and 44 to 77 years who underwent bilateral reduction mammaplasty for macromastia from 2016 through 2019. Only patients who met the criteria for surgically necessary, noncosmetic breast reduction surgery for the treatment of macromastia (using an inferior pedicle technique for breast reduction and Wise pattern nipple-preserving technique) were included in the study (Figure 1).^13^ Patients who were active smokers and patients with a history of breast malignancy (including incidental malignancy found on reduction mammaplasty pathology specimens), previous breast surgery or biopsy, or diabetes mellitus types 1 or 2 were excluded from the study. Data extraction was from standardized provider-entered consultation, perioperative, and follow-up clinic notes, containing patient symptomatology, clinical findings, photographs, and physical examination characteristics. All patients were evaluated in clinic during postoperative visits at the following intervals: 1 week, 1 month, 3 months, 6 months, and 12 months. The postoperative complications included for analysis in our sample are listed and defined in Figure 2.^14^

### Data Storage and Management

All patient data were collected from the Epic (Epic Systems Corporation) electronic medical record system. Deidentified patient data including demographic information, diagnosis of macromastia, complications related to wound healing, and postoperative complications were collected and stored in a secure computer database.

### Statistical Analysis

Patient demographic characteristics are summarized using descriptive statistics (mean ± standard deviation for continuous variables and frequencies and percentages for categorical variables). Significance was calculated using Fisher exact test/Pearson chi-squared test or *t* test measured with 2 tails, and *P* value significance was defined as ≤0.05.

## RESULTS

### Patient Demographic Information

Between 2016 and 2019, 308 patients underwent reduction mammaplasty at our institution. Of those, 141 patients met our inclusion criteria: 49 patients in the adolescent group and 92 patients in the average-age group (Table 1). Ages of the patients in the adolescent group ranged from 13 to 24 years, with a mean age of 19 ± 3.2 years at the time of surgery. Ages of the patients in the average-age group ranged from 44 to 77 years, with a mean age of 53 ± 7.4 years at surgery. Both groups were composed principally of African American patients: 55.1% in the adolescent group and 67.4% in the average-age group. Patients in the adolescent group had a significantly higher mean preoperative body mass index compared to the average-age patients: 31.1 ± 4.2 kg/m^2^ vs 29.4 ± 5.1 kg/m^2^ (*P*=0.047).

**Table 1.** Patient Demographic Information, n=141

Variable	Adolescent Group, n=49	Average-Age Group, n=92	*P* Value
Age, years, mean ± SD	19 ± 3.2	53 ± 7.4	**<0.001**
Race/ethnicity			
African American	27 (55.1)	62 (67.4)	0.149
White	13 (26.5)	17 (18.5)	0.265
Hispanic	9 (18.4)	11 (12.0)	0.299
Asian	0	1 (1.1)	1
Other	0	1 (1.1)	1
Body mass index, kg/m^2^, mean ± SD	31.1 ± 4.2	29.4 ± 5.1	**0.047**
Overweight, 25-29.9 kg/m^2^	23 (46.9)	48 (52.2)	0.554
Obese, >30 kg/m^2^	26 (53.1)	44 (47.8)	0.554

Notes: Data are presented as n (%) unless otherwise indicated. Patients in the adolescent group were ages 13 to 24 years, and patients in the average-age group were 44 to 77 years. *P* values were calculated using *t* test or Fisher exact test/Pearson chi-squared test. Significant values are in bold text.

### Complications Occurring During the Immediate Postoperative Period

No patients in either group experienced severe immediate complications occurring during the same hospital stay, such as death, deep vein thrombosis, or pulmonary embolism. One patient in the adolescent group (2.0%) and 2 patients in the average-age group (2.2%) required same-day operative take-back because of the presence of an expanding hematoma (Table 2). None of those patients required hospital admission for inpatient monitoring, and all 3 patients were cleared for same-day discharge. No statistically significant difference in same-day operative take-back was found between the adolescent and average-age patients (*P*=1).

**Table 2.** Early Complications Present From the Immediate Postoperative Period to the 1-Month Follow-Up

Complication	Adolescent Group, n=49	Average-Age Group, n=92	*P* Value
Same-day operative take-back (expanding hematoma)	1 (2.0)	2 (2.2)	1
Necrosis	2 (4.1)	6 (6.5)	0.714
Nipple	0	3 (3.3)	0.552
Areola	1 (2.0)	2 (2.2)	1
Skin	1 (2.0)	1 (1.1)	1
Requiring excision of necrosis	1 (2.0)	3 (3.3)	1
Surgical site infection	0	1 (1.1)	1
Wound dehiscence at T junction	2 (4.1)	8 (8.7)	0.494
Requiring debridement	1 (2.0)	3 (3.3)	1
Requiring operative repair	0	2 (2.2)	0.543
Requiring excision of necrosis	0	1 (1.1)	1
Hematoma/seroma	3 (6.1)	5 (5.4)	1
Requiring drainage in clinic	1 (2.0)	1 (1.1)	1
Requiring operative drainage	1 (2.0)	2 (2.2)	1
Total early complications	8 (16.3)	22 (23.9)	0.294

Notes: Data are presented as n (%). Patients in the adolescent group were ages 13 to 24 years, and patients in the average-age group were 44 to 77 years. *P* values were calculated using Fisher exact test/Pearson chi-squared test.

### Early Complications Present From the Immediate Postoperative Period to the 1-Month Follow-Up

Early minor complications related to wound healing occurred in both groups, but no significant differences were seen between adolescent and average-age patients for any complications occurring from the immediate postoperative period to the 1-month follow-up (Table 2).

Wound dehiscence at the T junction occurred in 4.1% of the adolescent group vs 8.7% of the average-age group, and hematoma/seroma occurred in 6.1% of the adolescent group vs 5.4% of the average-age group. Early surgical site infection occurred in 1 patient in the average-age group and was successfully treated with oral antibiotic therapy.

Of the 2 adolescent patients with wound dehiscence at the T junction site, 1 patient (50%) failed to achieve wound closure by the 1-month follow-up and required debridement in clinic with excision of skin necrosis; the other patient was managed with dressing changes and careful at-home monitoring. Of the 8 patients in the average-age group with an open T junction, 3 (3.3%) required debridement in clinic, and 2 (2.2%) required repair of the T junction site in the operating room, 1 of whom required excision of skin necrosis.

No nipple necrosis was seen in the adolescent group vs in 3 patients (3.3%) in the average-age group who developed nipple necrosis by the 1-month follow-up; all 3 patients required operative excision of necrosis. Of the 3 patients in the adolescent group who developed a hematoma/seroma persisting to the 1-month follow-up visit, 1 patient underwent successful aspiration in the clinic, and another patient underwent successful evacuation in the operating room. Of the 5 average-age patients with hematoma formation persisting to the 1-month follow-up visit, 1 patient underwent successful drainage in the clinic, and 2 patients had successful evacuations in the operating room.

### Midterm Complications Present at the 3-Month to the 6-Month Follow-Up

Midterm complications present during the 3- to 6-month follow-up period for both groups are shown in Table 3. No significant differences were seen between adolescent and average-age patients for any complications occurring during this time period.

**Table 3.** Midterm Complications Present at the 3-Month to the 6-Month Follow-Up

Complication	Adolescent Group, n=49	Average-Age Group, n=92	*P* Value
Nipple hypersensitivity	5 (10.2)	8 (8.7)	0.767
Unilateral	3 (6.1)	5 (5.4)	1
Bilateral	2 (4.1)	3 (3.3)	1
Nipple numbness	0	1 (1.1)	1
Unilateral	0	1 (1.1)	1
Nipple hypopigmentation	0	3 (3.3)	0.552
Unilateral	0	3 (3.3)	0.552
Nipple/breast cyst	0	1 (1.1)	1
Hypertrophic scarring/keloids	14 (28.6)	31 (33.7)	0.386
Total midterm complications	19 (38.8)	44 (47.8)	0.303

Notes: Data are presented as n (%). Patients in the adolescent group were ages 13 to 24 years, and patients in the average-age group were 44 to 77 years. *P* values were calculated using Fisher exact test/Pearson chi-squared test.

Five patients (10.2%) in the adolescent group had nipple hypersensitivity compared to 8 patients (8.7%) in the average-age group. No adolescent patients developed numbness of the nipple, while 1 patient (1.1%) in the average-age cohort did. Hypertrophic scarring/keloids occurred in 28.6% of adolescent patients vs 33.7% of average-age patients.

### Late Complications Present at the 12-Month Follow-Up

Late postoperative complications present at the 12-month follow-up visit in both groups were persisting nipple hypersensitivity, persisting hypertrophic scarring/keloids, and breast asymmetry (Table 4). No significant differences occurred in long-term complications between the adolescent and average-age groups.

**Table 4.** Late Complications Present at the 12-Month Follow-Up

Complication	Adolescent Group, n=49	Average-Age Group, n=92	*P* Value
Nipple hypersensitivity	1 (2.0)	1 (1.1)	1
Unilateral	1 (2.0)	1 (1.1)	1
Nipple hypopigmentation	0	1 (1.1)	1
Unilateral	0	1 (1.1)	1
Hypertrophic scarring/keloids	11 (22.4)	20 (21.7)	0.923
Breast asymmetry	4 (8.2)	5 (5.4)	0.719
Breast asymmetry requiring reoperation (revision mammaplasty)	0	1 (1.1)	1
Total late complications	16 (32.7)	28 (30.4)	0.787

Notes: Data are presented as n (%). Patients in the adolescent group were ages 13 to 24 years, and patients in the average-age group were 44 to 77 years. *P* values were calculated using Fisher exact test/Pearson chi-squared test.

### Total Complications Related to Wound Healing

Total complications related to wound healing are listed in Table 5. Complications related to wound healing occurred in 42.9% of adolescent patients vs 50.0% of average-age patients (*P*=0.418).

**Table 5.** Total Complications Related to Wound Healing

Complication	Adolescent Group, n=49	Average-Age Group, n=92	*P* Value
Wound dehiscence at T junction	2 (4.1)	8 (8.7)	0.494
Hematoma/seroma	3 (6.1)	5 (5.4)	1
Nipple hypersensitivity	5 (10.2)	8 (8.7)	0.767
Nipple numbness	0	1 (1.1)	1
Nipple hypopigmentation	0	3 (3.3)	0.552
Nipple/breast cyst	0	1 (1.1)	1
Hypertrophic scarring/keloids	11 (22.4)	20 (21.7)	0.923
Total complications related to wound healing	21 (42.9)	46 (50.0)	0.418

Notes: Data are presented as n (%). Patients in the adolescent group were ages 13 to 24 years, and patients in the average-age group were 44 to 77 years. *P* values were calculated using Fisher exact test/Pearson chi-squared test.

### Total Postoperative Complications

Total postoperative complications excluding those related to wound healing (refer to Figure 2), are listed in Table 6. Postoperative complications occurred in 18.4% of adolescent patients vs 19.6% of average-age patients (*P*=0.863).

**Table 6.** Total Postoperative Complications

Complication	Adolescent Group, n=49	Average-Age Group, n=92	*P* Value
Necrosis	2 (4.1)	6 (6.5)	0.714
Nipple	0 (0)	3 (3.3)	0.552
Areola	1 (2.0)	2 (2.2)	1
Skin	1 (2.0)	1 (1.1)	1
Surgical site infection	0 (0)	1 (1.1)	1
Reoperation and procedures	3 (6.1)	6 (6.5)	1
Operative take-back	2 (4.1)	2 (2.2)	0.610
Excision of necrosis	1 (2.0)	4 (4.3)	0.658
Breast asymmetry	4 (8.2)	5 (5.4)	0.719
Total postoperative complications	9 (18.4)	18 (19.6)	0.863

Notes: Data are presented as n (%). Patients in the adolescent group were ages 13 to 24 years, and patients in the average-age group were 44 to 77 years. *P* values were calculated using Fisher exact test/Pearson chi-squared test.

## DISCUSSION

Reduction mammaplasty is an established method for treating the negative symptoms of macromastia.^1,2,5,6^ However, in young and adolescent females, the decision to operate is often influenced by factors such as complication risk and the effects on breastfeeding and regrowth of breast tissue.^11,15,16^ Our study only examined complications in patients after undergoing reduction mammaplasty; to our knowledge, this study is the first to directly compare the outcomes of adolescent vs average-age patients at the same institution.

### Complication Rates

In our study population, minor complications were common in both age groups and mirror reports in the literature.^7,8,11,12^ To date, no standard definitions for complication classifications exist for reduction mammaplasty which results in a wide range of reported rates. For example, in the retrospective study by Winter et al,^17^ postoperative complications were defined as “any deviation from the normal postoperative course,” according to the Clavien-Dindo Grade I surgical complication criteria.^18^^,^^19^ Consequently, wound healing problems such as dehiscence, changes in sensation, and hypertrophic scarring were included in the calculation, resulting in an overall complication rate of 63%.^17^ Meanwhile, in their multicenter analysis, Gust et al only included major complications, thus reporting an overall complication rate of 4%.^20^ The systematic review by Fairchild et al included major complications plus adverse events, with wound dehiscence included but not hypertrophic scarring, cysts, or altered nipple sensation/pigmentation, resulting in an overall complication rate of 5%.^21^

### Optimal Timing and Breast Regrowth

In adolescent females, the optimal age for reduction mammaplasty remains unclear. Alleviating somatic and psychosocial symptoms should be weighed against the possibility of postoperative breast growth in young females.^15,16^ Nuzzi et al reported regrowth occurring in 6.1% of adolescents; approximately half was attributable to glandular growth and the other half to weight gain.^15^ While the role of postoperative weight gain and breast regrowth is not completely understood, the amount of initial breast tissue removed is strongly correlated to patient body mass index at surgery.^8,12^ In terms of female development, stabilization of breast growth is generally considered to occur 2 to 3 years after menarche; however, Nuzzi et al described obese patients reporting continued growth for an average of 9 years postmenarche.^15^ Xue et al showed that patients with a 1-year history of breast growth stabilization did not demonstrate any significant regrowth and recommended timing of surgery based on this principle, rather than waiting until adulthood.^16^ Although growing evidence suggests that progesterone-based contraception can cause glandular enlargement of breast tissue, a theorized cause of macromastia,^22^ Nuzzi et al reported that use of progestin-only contraception following reduction mammaplasty was not associated with breast regrowth.^23^

Brzozowski et al showed that the hormonal-driven breast enlargement via engorgement of ductal-lobular units remained intact in patients who become pregnant after reduction mammaplasty.^24^ However, no long-term studies have provided quantifiable data on the incidence rates of patients whose breast volumes return to post–reduction mammaplasty volumes after undergoing regrowth during breastfeeding.

### Effect on Breastfeeding

The data on breastfeeding success after reduction mammaplasty appear to be reassuring. In the Kraut et al systematic review of 51 studies, no significant impairment of breastfeeding ability was found in females who underwent breast reduction with preservation of the subareolar parenchyma (eg, superior, medial, or inferior pedicle/vertical reduction mammaplasty) or techniques that do not result in complete transection of the lactiferous ducts or lobular units.^25^ On the other hand, techniques that require free nipple grafting or transplant, a procedure that requires a complete transection of all lactiferous ducts, may likely reduce or remove the probability of future breastfeeding.^26-29^ Furthermore, the loss of nipple sensation, thought to be best preserved using an inferior pedicle technique,^30^ effectively blocks the nervous system reflex arc to the pituitary gland, inhibiting the release of prolactin and oxytocin and thus preventing the production and let-down of breast milk.^31-33^ Nonetheless, the consensus from the literature indicates that females who undergo pedicled breast reduction show no significant difference in breastfeeding ability compared to females without a history of reduction mammaplasty.^25-30^

### Limitations

The results of our study are subject to the statistical limitations of a retrospective review with a moderate sample size of 141 patients, thus creating the possibility for bias.

## CONCLUSION

Minor complications are common in reduction mammaplasty, although rates of life-threatening complications are rare. In this 3-year review comparing the outcomes of adolescent vs average-age patients who underwent reduction mammaplasty at the same institution, we found no significant differences in rates of postoperative complications. Our data suggest that adolescent patients with macromastia should not defer reduction mammaplasty out of concern for higher complication rates attributable to age alone.

## Notes

*This article meets the Accreditation Council for Graduate Medical Education and the American Board of Medical Specialties Maintenance of Certification competencies for Patient Care and Medical Knowledge.*
